# The brain-gut axis and chronic pain: mechanisms and therapeutic opportunities

**DOI:** 10.3389/fnins.2025.1545997

**Published:** 2025-02-14

**Authors:** Tim Ho, Ömer Elma, Lucy Kocanda, Katherine Brain, Thao Lam, Tejas Kanhere, Huan-Ji Dong

**Affiliations:** ^1^Faculty of Medicine and Health, Sydney Medical School, Westmead Clinical School, The University of Sydney, Sydney, Australia; ^2^Unit of Physiotherapy, Department of Rehabilitation and Sport Sciences, Faculty of Health and Social Sciences, Bournemouth University, Bournemouth, United Kingdom; ^3^Pain in Motion International Research Group, Vrije Universiteit Brussels, Brussels, Belgium; ^4^Department of Rural Health, College of Health, Medicine and Wellbeing, University of Newcastle, Tamworth, NSW, Australia; ^5^Tamworth Integrated Pain Service, Hunter New England Local Health District, Tamworth, NSW, Australia; ^6^Food and Nutrition Research Program, Hunter Medical Research Institute, New Lambton Heights, NSW, Australia; ^7^Hunter Integrated Pain Service, Hunter New England Local Health District, New Lambton Heights, NSW, Australia; ^8^School of Health Sciences, College of Health, Medicine and Wellbeing, University of Newcastle, Callaghan, NSW, Australia; ^9^Faculty of Medicine and Health, University of New South Wales, Sydney, Australia; ^10^Pain and Rehabilitation Centre, and Department of Health, Medicine and Caring Sciences, Linköping University, Linköping, Sweden

**Keywords:** chronic pain, brain-gut axis, microbiome, vagus nerve, HPA–hypothalamic-pituitary-adrenal

## Abstract

The brain-gut axis (BGA) is emerging as a critical mediator in chronic pain, involving bidirectional communication between the central nervous system and the gastrointestinal system. The “Pain Matrix” is associated with microbial dysbiosis, vagus nerve dysfunction, and hypothalamic-pituitary-adrenal (HPA) axis dysregulation, driving neuroinflammation and central sensitization. Key mechanisms include microbial diversity loss, leaky gut, and altered neuroactive signaling via short-chain fatty acids (SCFAs) and vagal pathways. This narrative review explores the intricate interplay between BGA mechanisms and chronic pain, highlighting therapeutic opportunities such as restoring dysbiosis, modulating vagus nerve activity, and regulating endocrine pathways. These interventions target inflammation, autonomic balance, and stress/reward pathway modulation, offering a promising path toward integrative pain management. Further research is required to validate these strategies and improve patient outcomes.

## 1 Introduction

Chronic pain is defined as pain persisting or recurring for longer than 3 months (Raffaeli et al., 2021). It is conceptualized as a long-term condition influenced by central nervous system mechanisms, as well as psychosocial factors (Gatchel et al., 2007). Chronic pain management requires an integrative, multidisciplinary approach, including pharmacological treatments, psychological support, physical rehabilitation, and lifestyle modifications to address its complex pathophysiology and common comorbidities. This complexity results in the limited effectiveness of pharmacological therapies, with side effects being extremely common (Martel et al., 2015). Specifically, the chronic primary pain discussed in this paper refers to pain that persists for more than three months, and is associated with significant emotional distress or functional disability, and cannot be better explained by another condition associated according to the International Association Study of Pain classification (Nicholas et al., 2019).

It is well-known that the gut and brain have a bidirectional communication pathway, and brain-gut interactions play an important role in regulating vital functions in the human body (Mayer and Tillisch, 2011). The gut has been called the “second brain” and connects the vagus nerve and brainstem, via spinal afferents in the spinal cord. Research over the past decade has significantly advanced our understanding of brain-gut interactions in gastrointestinal diseases and abdominal pain syndromes (e.g., irritable bowel syndrome and functional dyspepsia) (Guo et al., 2019). Evidence shows that sensitization mechanisms play a role in chronic pain conditions such as fibromyalgia, headaches, and neuropathic pain. These mechanisms are closely linked to brain-gut axis dysregulation, highlighting the need for targeted treatments (Guo et al., 2019). Several informative reviews have provided extensive knowledge on animal studies (Li et al., 2019; Pak et al., 2024; Rusch et al., 2023).

This narrative review focuses on the understanding of the factors affecting alterations in brain-gut axis (BGA) related to peripheral and central sensitization. Understanding these underlying mechanisms is essential for developing therapeutic approaches in integrative pain management.

## 2 Materials and methods

### 2.1 Research question

This narrative review addressed the following question: What are the underlying mechanisms of interaction between the brain-gut axis (BGA) and chronic primary pain?

### 2.2 Eligibility criteria

Studies eligible for inclusion in the review included:

• Populations with chronic primary pain conditions based on International Classification of Diseases (ICD) 11 (e.g., fibromyalgia, irritable bowel syndrome [IBS], chronic pelvic pain, other nociplastic pain) in human subjects. Animal studies were excluded.
• Examination of mechanistic insights into the BGA, including microbial diversity, inflammation, gut barrier integrity, neuroendocrine modulation, vagal nerve activation, and autonomic nervous system (ANS) or hypothalamic-pituitary-adrenal (HPA) axis involvement. Pharmacological intervention studies were excluded.
• Systematic reviews, narrative reviews, clinical research (e.g., observational studies), and basic science investigations. Non-peer-reviewed materials (e.g., opinion articles, letters, and conference abstracts) and articles published before 2000 or in languages other than English were excluded.

### 2.3 Data sources

The literature search included the following databases to ensure comprehensive coverage:

• PubMed
• Web of Science
• Google Scholar
• EBSCO

### 2.4 Search strategy

A structured search strategy was employed to identify relevant studies. The search terms included combinations of the following keywords (Table 1):

**TABLE 1:** Search terms for PICO framework.

PICO component	Search terms
Population (P)	“chronic pain,” “chronic primary pain,” “fibromyalgia,” “IBS,” “chronic pelvic pain,” “nociplastic pain”
Intervention (I)	“gut-brain axis,” “microbiome diversity,” “gut barrier,” “vagal nerve,” “HPA axis,” “SCFAs,” “dysbiosis”
Comparison (C)	“healthy controls,” “normal microbiota,” “placebo,” “sham interventions”
Outcome (O)	“pain modulation,” “neuroinflammation,” “dysbiosis,” “autonomic imbalance”

Boolean operators (AND, OR) were used to combine search terms, and the search strategy was adapted for each database to optimize retrieval of relevant literature.

### 2.5 Study selection

The identified studies underwent a structured screening process to ensure relevance:

• Initial screening: Titles and abstracts were reviewed against the eligibility criteria to exclude irrelevant studies.
• Full-text review: Studies that passed the initial screening were reviewed in full to confirm inclusion. Conflicts during the screening process were resolved through discussion or by consulting a third reviewer.

### 2.6 Data organization and presentation

Findings from the included studies were synthesized narratively, with a focus on thematic organization. Key mechanisms underlying the interaction between the brain-gut axis (BGA) and chronic primary pain were highlighted, including:

• The role of microbial diversity, inflammation, and gut barrier integrity.
• Neuroendocrine modulation, vagal nerve activation, and autonomic nervous system (ANS) involvement.
• The hypothalamic-pituitary-adrenal (HPA) axis and its contribution to chronic pain mechanisms.

## 3 Microbiome diversity and chronic pain: connected via brain-gut axis

Understanding microbial diversity within the brain-gut axis is essential for grasping its implications for chronic pain. Microbial diversity is generally assessed through alpha and beta diversity indices. Alpha diversity evaluates within-individual diversity using metrics like Shannon, Simpson, and Faith’s phylogenetic diversity, while beta diversity examines differences across individuals or groups (Freidin et al., 2020; Goudman et al., 2024; Li et al., 2022; Liu et al., 2023).

### 3.1 Alpha diversity, species-level diversity and pain pathways

In the context of chronic pain, alpha diversity metrics—especially Shannon and Faith’s indices—have been associated with neuroinflammatory processes. Although not all studies target chronic pain specifically, reduced alpha diversity is linked to neuroinflammatory and neurological conditions, suggesting parallels with pain sensitization mechanisms (Goudman et al., 2024). Certain taxa, such as *Coprococcus comes* and *Faecalibacterium prausnitzii*, known for anti-inflammatory roles, are often depleted in chronic pain. Additionally, butyrate-producing bacteria like those in the Lachnospiraceae family support gut health and inflammation modulation, indicating their potential impact on pain pathways (Freidin et al., 2020; Goudman et al., 2024).

While broader taxonomic diversity offers insights into overall composition, species-level diversity—such as *Faecalibacterium prausnitzii* and *Odoribacter splanchnicus* abundance—provides specific relevance to chronic pain. These bacteria produce anti-inflammatory metabolites, such as butyrate, which enhance gut barrier integrity, mitigate systemic inflammation, and modulate neuroinflammatory pathways implicated in pain sensitization. However, current data limitations underscore the need for further species-level research to clarify these associations with neuroinflammatory outcomes (Freidin et al., 2020; Liu et al., 2023).

### 3.2 Mechanisms of communication with the central nervous system

Communication of microbial diversity changes to the central nervous system (CNS) occurs through gut barrier integrity, immune signaling, metabolites, and neurotransmitters. Increased intestinal permeability, or “leaky gut,” permits microbial products like lipopolysaccharides (LPS) into circulation, activating gut-associated lymphoid tissue (GALT) and leading to neuroinflammation that may heighten pain sensitivity. Microbial metabolites, especially short-chain fatty acids (SCFAs) like butyrate, fortify gut barrier integrity and reduce pro-inflammatory cytokines, indirectly protecting the CNS. Microbial neurotransmitters, including GABA and serotonin, interact with the CNS via the vagus nerve, influencing mood and pain perception. Dysbiosis in these pathways reinforces the brain-gut axis as a significant target for chronic pain management (Freidin et al., 2020; Li et al., 2022; Liu et al., 2023).

Table 2 outlines the key microbiota species identified in chronic pain conditions, highlighting observed changes in their abundance and their potential mechanistic roles. The table categorizes microbiota at the species level, showing reductions or increases in specific taxa across various chronic pain disorders, such as migraine, fibromyalgia, and bladder pain syndrome. The mechanisms discussed include their roles in anti-inflammatory processes (e.g., butyrate production), gut barrier maintenance, and modulation of neuroinflammatory pathways, illustrating their relevance as potential targets for intervention in chronic pain management.

**TABLE 2:** Key microbiota species identified in chronic pain conditions and conditions associated with chronic pain symptoms.

Microbiota species (Freidin et al., 2020; Goudman et al., 2024)	Observation in chronic pain	Potential mechanism
*Faecalibacterium prausnitzii*	Decreased in migraine, fibromyalgia, and ME/CFS patients.	Anti-inflammatory via butyrate production; supports gut barrier integrity.
*Coprococcus catus*	Reduced in ME/CFS patients.	SCFA producer; supports gut and CNS homeostasis.
*Coprococcus comes*	Reduced in chronic pain conditions like ME/CFS.	SCFA producer; contributes to anti-inflammatory signaling.
*Ruminococcus obeum*	Reduced in ME/CFS and migraine patients.	SCFA producer; role in maintaining gut health.
*Odoribacter splanchnicus*	Reduced in bladder pain syndrome, migraine, and ME/CFS.	Produces SCFAs; modulates gut immune responses.
*Roseburia inulinivorans*	Decreased in fibromyalgia and chronic fatigue syndrome.	Produces butyrate; enhances gut epithelial integrity.
*Eggerthella lenta*	Increased in migraine and ME/CFS patients.	Pro-inflammatory activity; alters gut-brain communication.
*Clostridium symbiosum*	Increased in migraine and ME/CFS patients.	Produces metabolites impacting gut-brain signaling.

## 4 The role of the vagus nerve in brain-gut axis and pain modulation

The vagus nerve, the tenth and longest cranial nerve, plays a pivotal role in the modulation of various physiological processes, including pain perception, inflammation, and homeostasis. With its extensive afferent and efferent projections connecting the brainstem to visceral organs, the vagus nerve is a critical component of the brain-gut axis and the autonomic nervous system (ANS).

### 4.1 Neural pathways connecting the vagus nerve to central pain networks

The vagus nerve connects to central pain networks primarily through its afferent fibers, which transmit sensory information from visceral organs to the brainstem. The nucleus tractus solitarius (NTS) serves as a primary relay station, where afferent vagal signals are integrated and relayed to other brain regions, including the locus coeruleus, hypothalamus, and amygdala. These areas are involved in the modulation of pain, emotional responses, and autonomic functions (Bonaz et al., 2017).

Through its connections with the locus coeruleus, the vagus nerve can influence the release of norepinephrine, which plays a key role in the descending pain inhibitory pathways. Additionally, projections from the NTS to the periaqueductal gray (PAG) and raphe nuclei activate serotonergic and opioid pathways, further contributing to pain inhibition (Morris et al., 2020).

This intricate network underscores the vagus nerve’s capacity to modulate pain perception both directly and indirectly.

### 4.2 Chronic pain and plastic changes in the vagus nerve

Chronic pain is associated with significant alterations in the neural pathways and structures involved in pain perception and modulation. The vagus nerve, due to its extensive connections with the brainstem and spinal cord, undergoes plastic changes in response to chronic pain (Wu et al., 2024). These changes involve the sensitization of nociceptive pathways and the alteration of autonomic regulation. Specifically, chronic pain enhances the activity of the NTS, (Shao et al., 2023) which is a critical hub for afferent vagal fibers. This heightened activity can lead to increased sympathetic output and altered parasympathetic tone, further perpetuating pain and stress responses.

The implications of these plastic changes are profound. By modulating the vagus nerve, it may be possible to reverse or attenuate these maladaptive alterations, thereby reducing pain perception and improving autonomic balance. For instance, vagus nerve stimulation (VNS) has been shown to decrease the activity of nociceptive neurons in the spinal cord and brainstem, potentially reversing the hyperexcitability associated with chronic pain (Shao et al., 2023).

### 4.3 Neuroactive molecules, short-chain fatty acids and vagus nerve activity in chronic primary pain conditions

In patients with chronic primary pain conditions (e.g., fibromyalgia, low back pain, etc.), levels of neuroactive molecules related to vagus nerve activity, such as norepinephrine, serotonin, and acetylcholine, are often dysregulated. Studies have shown that these patients exhibit altered levels of these molecules compared to healthy controls, and these alterations correlate with pain severity and brain connectivity (Yang and Chang, 2019).

Short-chain fatty acids (SCFAs), particularly acetate, propionate, and butyrate, are produced by gut microbiota through the fermentation of dietary fibers. These SCFAs play a crucial role in communicating with the brain via the vagus nerve, forming an essential part of the gut-brain axis. SCFAs can directly activate the vagus nerve, with butyrate shown to increase the firing rate of vagal afferent neurons. The SCFA receptor FFAR3, expressed on vagal afferents, is shown to be important for this communication (Mansuy-Aubert and Ravussin, 2023).

In addition, other receptors such as GPR109A and OR51E2 mediate SCFA effects. GPR109A, expressed on immune cells like macrophages and dendritic cells, is activated by butyrate and plays a key role in anti-inflammatory processes and metabolic regulation. While there is no direct evidence that GPR109A regulates the vagus nerve, its role in reducing inflammation and maintaining gut homeostasis may indirectly influence vagal activity through the gut-brain axis. Similarly, OR51E2, another SCFA receptor expressed on immune cells, senses acetate and propionate and indirectly affects vagus nerve activity by modulating immune responses (Mansuy-Aubert and Ravussin, 2023; O’Riordan et al., 2022).

### 4.4 Vagus nerve modulation of inflammation via the gut barrier and spleen

The vagus nerve has an efferent activity that may modulate inflammation through its interactions with the gut barrier and the spleen. This modulation is primarily mediated by the cholinergic anti-inflammatory pathway (CAIP), which involves the release of acetylcholine from efferent vagal fibers. Acetylcholine binds to receptors on macrophages in the spleen, inhibiting the release of pro-inflammatory cytokines such as TNF-α, IL-1, and IL-6. In addition to its direct anti-inflammatory effects, the vagus nerve influences the integrity of the gut barrier. By modulating gastrointestinal motility and secretion, the vagus nerve helps maintain the balance of the gut microbiota, which is critical for preventing the translocation of pathogens and endotoxins into the bloodstream. This gut-brain communication is essential for controlling systemic inflammation and maintaining immune homeostasis (Breit et al., 2018).

## 5 Endocrine pathways in the gut brain axis

The gut-brain axis also includes bidirectional hormonal signaling pathways aimed at relaying information about ingested nutrients during a meal to the brain, which integrates these signals to coordinate the regulation of food intake, energy expenditure and glucose homeostasis via enteroendocrine cells within the intestinal epithelium secreting hormones such as GLP-1 and cholecystokinin.

### 5.1 Role of GLP-1 in pain modulation

Indeed, GLP-1 has been shown in pre-clinical models to modulate chronic pain by reducing neuroinflammation, enhancing anti-inflammatory mediators (e.g., IL-10, β-endorphins), and regulating pain signaling in the dorsal horn. In preclinical studies, GLP-1 also modulates brain circuitry by reducing neuroinflammation, altering dopaminergic activity, and decreasing reward sensitivity in regions like the nucleus accumbens and ventral tegmental area (Halloum et al., 2024; Martinelli et al., 2024; Wachsmuth et al., 2022).

### 5.2 The hypothalamic-pituitary-adrenal axis and chronic cortisol dysregulation

The hypothalamic-pituitary-adrenal (HPA) is a major hormonal system within the body linked with stress response and is thought to play a key role in regulating the brain-gut axis (Dinan and Cryan, 2016). Hormones within the HPA axis include Corticotrophin Releasing Hormone (CRH) released by the hypothalamus, which stimulates the pituitary gland to release Adrenocorticotropic Hormone (ACTH), which then triggers the adrenal glands to produce cortisol. Elevated cortisol levels are associated with alteration of gut microbiota composition as well as increased gut permeability and this has been linked to impaired neural activity and connectivity, often resulting in cognitive, emotional, and stress regulation deficits (Rusch et al., 2023). ACTH is also thought to modulate the inhibitory controls of endogenous opioid peptides, which can influence pain processing (Hannibal and Bishop, 2014).

The HPA axis, a key regulator of the stress response, is frequently dysregulated in chronic pain, marked by sustained cortisol release due to impaired negative feedback in the hypothalamus and pituitary. This prolonged cortisol elevation perpetuates a maladaptive stress response, contributing to persistent pain. Chronic high cortisol levels are linked to reduced hippocampal volume, amplifying stress reactivity and reinforcing pain sensitivity, creating an allostatic load that perpetuates a harmful feedback loop in pain perception and stress response (Rusch et al., 2023; Vachon-Presseau et al., 2013).

Unlike the temporary anti-inflammatory effects of acute cortisol release, chronic cortisol elevation induces a pro-inflammatory state. Tissues develop resistance to cortisol, blunting its anti-inflammatory role and leading to cytokine release that sensitizes pain pathways. In the CNS, this promotes neuroinflammation, particularly in areas like the parahippocampal gyrus, intensifying pain and anxiety-related response (Hannibal and Bishop, 2014; Rusch et al., 2023).

### 5.3 Impact on gut integrity and systemic inflammation

HPA axis dysregulation impacts gut integrity by increasing gut permeability (“leaky gut”), allowing microbial products to enter the bloodstream and trigger systemic inflammation. This inflammation can further activate the CNS, linking gut health with pain perception and explaining the association of chronic pain with gastrointestinal symptoms, such as irritable bowel syndrome (IBS) (Rusch et al., 2023).

## 6 Future directions and emerging research in brain-gut axis modulation for chronic pain management

Emerging research on the brain-gut axis (BGA) offers promising avenues for innovative chronic pain therapies (Mayer and Tillisch, 2011; Pak et al., 2024). This section highlights several key areas for future investigation, focusing on microbiome communication with the brain, vagus nerve function and its modulation, and endocrine pathways relating to BGA. By advancing research across these domains, we can deepen our understanding of the brain-gut axis and its role in chronic pain, moving toward holistic, innovative treatments.

### 6.1 Understanding microbiome communication with the brain in pain modulation

Microbial diversity and its communication with the central nervous system (CNS) through metabolites and neurotransmitters presents an exciting research frontier. Reduced microbial diversity—especially a loss of anti-inflammatory bacteria like *Faecalibacterium prausnitzii*—is associated with neuroinflammation and heightened pain sensitivity (Freidin et al., 2020; Goudman et al., 2024). Future studies should expand species-level analyses to uncover microbial taxa that specifically influence pain pathways, as well as evaluate how microbial metabolites like butyrate interact with vagal and CNS receptors, such as FFAR3, which is implicated in gut-brain communication (Mansuy-Aubert and Ravussin, 2023). Longitudinal studies assessing the effects of probiotics, dietary interventions, and fecal microbiota transplantation (FMT) on microbial diversity and chronic pain outcomes could further validate microbiome modulation as a therapeutic approach.

### 6.2 The role of GLP-1 receptor agonists

Glucagon-like peptide-1 receptor (GLP-1R) agonists present a promising avenue for chronic pain management, as preclinical studies demonstrate their role in modulating neuroinflammation, central sensitization, and pain signaling pathways. These agents act on GLP-1Rs expressed in microglial cells and spinal neurons to attenuate inflammatory and neuropathic pain by reducing pro-inflammatory cytokines and promoting anti-inflammatory mediators such as interleukin-10. Additionally, GLP-1R agonists reduce hypersensitivity without inducing tolerance, offering potential for sustained analgesia (Halloum et al., 2024). Further research is needed to clarify mechanisms, validate efficacy in human trials, and explore combinations with other therapies to optimize outcomes in pain management.

### 6.3 Vagus nerve modulation and neuroplasticity

The vagus nerve’s role in autonomic and inflammatory regulation highlights its potential as a therapeutic target for chronic pain management. Vagus nerve stimulation (VNS), both invasive and non-invasive, has shown promise in reducing pain and improving autonomic function (Shao et al., 2023). Future research should investigate how VNS impacts neuroplasticity in pain pathways and influences markers of autonomic function, such as heart rate variability (HRV). Elucidating the neural adaptations resulting from VNS may help refine treatment protocols for chronic pain conditions with autonomic dysregulation, like fibromyalgia and irritable bowel syndrome (IBS).

**FIGURE 1:**
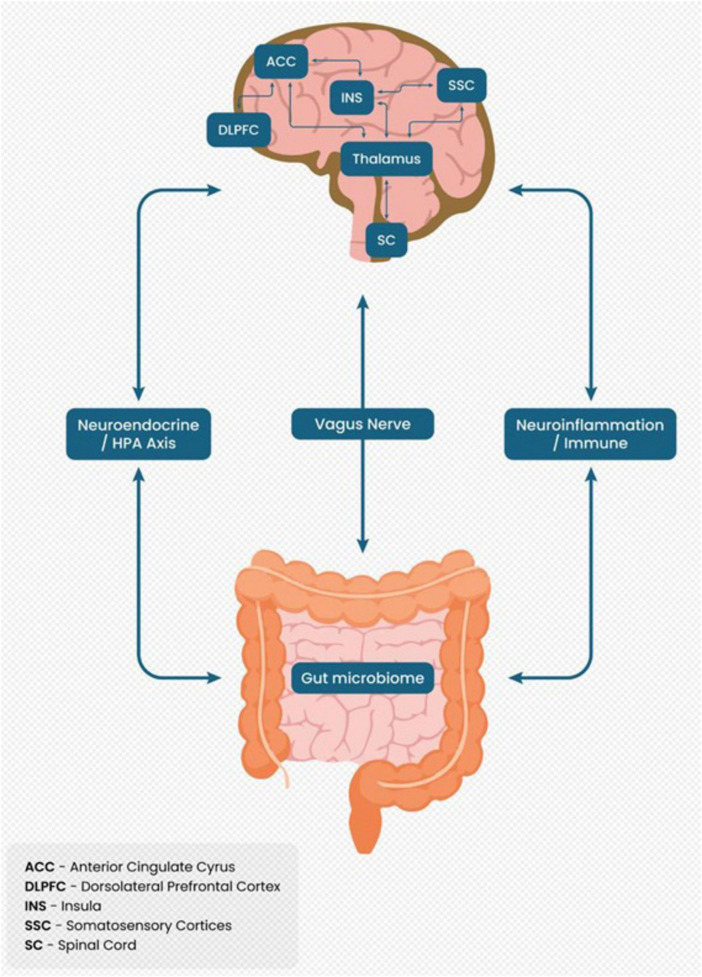
Brain-gut axis as a bidirectional communication system in chronic pain.

Figure 1 illustrates the BGA as a bidirectional communication system. It highlights connections between the gut microbiome, vagus nerve, neuroendocrine pathways (HPA axis), and CNS regions such as the anterior cingulate cyrus, insula, thalamus, and spinal cord, emphasizing their role in chronic pain mechanisms.

Table 3 details the mechanisms and potential therapeutic targets within the BGA. It covers categories such as the microbiome, inflammation, vagal nerve, and endocrine pathways, linking these mechanisms to emerging interventions like probiotics, SCFA supplementation, VNS, and GLP-1 receptor agonists for chronic pain management.

**TABLE 3:** Mechanisms and therapeutic targets in the brain-gut axis for chronic pain.

Category	Key mechanisms	Potential targets for future research
Microbiome	Reduced diversity (e.g., Faecalibacterium depletion) linked to neuroinflammation.	Probiotics, dietary interventions, FMT to enhance microbial diversity.
	SCFAs (e.g., butyrate) support gut barrier and reduce inflammation.	SCFA supplementation or microbiota modulation therapies.
	Dysbiosis alters neurotransmitter signaling (e.g., GABA, serotonin).	Microbiota-targeting interventions to influence CNS signaling pathways.
Inflammation	Leaky gut and cytokines (e.g., TNF-α) drive neuroinflammatory sensitization.	Therapies to strengthen gut barrier integrity and reduce systemic cytokines.
Vagal nerve	Links gut to CNS pain centers (e.g., NTS, PAG).	Non-invasive and invasive VNS for pain and autonomic dysfunction.
	SCFAs activate vagal pathways, influencing pain and inflammation.	Enhancing SCFA signaling to leverage vagal modulation.
Endocrine/HPA axis	Dysregulated cortisol elevates neuroinflammation and gut permeability.	Strategies to rebalance the HPA axis and reduce chronic stress responses.
	GLP-1 reduces neuroinflammation and modulates pain signaling.	Development of GLP-1 receptor agonists for chronic pain management.

## 7 Conclusion

The BGA represents a vital framework for understanding the mechanisms underlying chronic pain, highlighting the complex interplay between the gut microbiome, vagus nerve, and neuroendocrine pathways. Dysbiosis, neuroinflammation, and HPA axis dysregulation are key drivers of chronic pain, impacting gut integrity, autonomic regulation, and central sensitization. Therapeutic opportunities such as dietary interventions, microbiome modification, vagus nerve stimulation, and neuroendocrine regulation offer promising pathways for integrative management, targeting neuroinflammation, autonomic balance, stress response, and reward circuitry regulation.

Future research should focus on validating these interventions in clinical settings, enhancing our understanding of species-specific microbial roles, and refining neuromodulation techniques. Leveraging these insights can lead to more effective, holistic approaches to chronic pain management, ultimately improving patient outcomes.
